# FBXL20 acts as an invasion inducer and mediates E-cadherin in colorectal adenocarcinoma

**DOI:** 10.3892/ol.2014.2031

**Published:** 2014-04-03

**Authors:** JIANJUN ZHU, SHISHAN DENG, JIE DUAN, XINGGUO XIE, SHIQUAN XU, MAOCHENG RAN, XIAOSI DAI, YU PU, XIAOMING ZHANG

**Affiliations:** 1 Department of Human Anatomy, Premedical College, North Sichuan Medical College, Nanchong, Sichuan 637000, P.R. China; 2 Sichuan Key Laboratory of Medical Imaging, Nanchong, Sichuan 637000, P.R. China; 3 Department of Neurosurgery, Affiliated Hospital of North Sichuan Medical College, Nanchong, Sichuan 637000, P.R. China

**Keywords:** FBXL20, colorectal adenocarcinoma, E-cadherin, invasion

## Abstract

The mechanisms eliciting colorectal adenocarcinoma are not well understood and the FBXL20 gene is problematic as it exhibits an abnormal expression in colorectal cancer cells. In the present study a recombinant plasmid, pReceiver-M03-FBL20 expression plasmid was constructed, which overexpressed FBXL20; this was transfected into Lovo cells to form Lovo-FBL20 cells. The FBXL20 expression level was examined by quantitative polymerase chain reaction (qPCR) and western blot analysis. The cell viability and invasion capacity were measured using cell counting kit 8, Transwell chamber and wound healing assays, respectively. The associated genes, including E-cadherin, β-catenin, c-Myc, SET nuclear oncogene, protein phosphatase-2A, Axin, p53 and caspase 3, were detected by qPCR and western blotting. It was demonstrated that the FBXL20 expression level was markedly upregulated in the Lovo-FBL20 cells transfected with pReceiver-M03-FBL20 expression plasmid, compared with that of the Lovo cells. In addition, the cell viability and invasion capacity of the Lovo-FBL20 cells were significantly increased. These increases correlated with a significant upregulation in the expression level of β-catenin and c-Myc, and a downregulated expression level of E-cadherin. The results of the present study indicate that FBXL20 may mediate the ubiquitin degradation of E-cadherin resulting in an increased invasive ability of malignant cells.

## Introduction

Colorectal adenocarcinoma is one of the most common types of malignant tumor, with an increasing prevalence over recent years. It has the second highest incidence rate of all types of cancers and it is the second most common cause of cancer-associated mortality in Europe (1,2). Carcinogenesis of colorectal adenocarcinoma includes numerous genomic alterations and is a multi-step process. Thus far, a number of genes, including K-ras, DCC, p53, nm23, β-catenin, c-Myc, cyclin-dependent kinases and the caspase family, have been reported to be associated with the tumorigenesis and development of colorectal adenocarcinoma (3–6). However, the mechanism of tumorigenesis remains unclear. Therefore, the genes that may be associated with tumorigenesis require further investigation, as understanding the molecular mechanism of this process may aid with cancer prevention, early diagnosis and effective treatment.

The FBXL20 gene contains 10,381 base pairs (bps), with a 1,308-bp open reading frame. It is located on the human chromosome 17q21.2, and predicts to encode a 436-amino-acid protein that contains an F-box motif, which is the key feature of F-box proteins (FBPs). The essential role of FBPs has been demonstrated in studies of various types of species for ubiquitin-mediated degradation of cellular regulatory proteins. Welcker *et al* (7) reported that the F-box and WD repeat domain containing 7 (Fbw7) tumor suppressor, a member of the F-Box family, regulates glycogen synthase kinase 3 (GSK3) phosphorylation-dependent c-Myc protein degradation. Furthermore, c-Myc proteins regulate cell growth and division in numerous types of human cancer. The study showed that phosphorylation of c-Myc on threonine-58 by GSK3 regulates the binding of Fbw7 to c-Myc. Therefore, the activation of c-Myc is a significant oncogenic consequence of the loss of Fbw7 in cancer (7). In another study, it was shown that the accumulation of cyclin-dependent kinase inhibitor, p27 was caused by S-phase kinase-associated protein 2, another member of the F-Box family, and the upregulated p27 level may be a good indicator of proliferative activity and poor prognosis (8). Similar types of studies have been carried out to investigate the structure and function of the F-Box family members. These studies demonstrated that the F-Box family members are significant in tumorigenesis and development by inducing the specific targeting proteins into the ubiquitin proteasome process.

Our previous study on FBXL20 showed that the gene was critical in the abnormal Wnt signaling pathway, as the β-catenin expression level was significantly decreased after silencing the FBXL20 gene in the colon adenocarcinoma SW480 and SW620 cells (9). It was also identified that FBXL20 was potentially involved in the ubiquitin-mediated degradation process of E-cadherin and the SET nuclear oncogene. The viability of the colon cells, that transfected small interfering RNA targeted to the FBXL20 gene, was significantly inhibited. In addition, the marked increase of the E-cadherin expression level and the significant decrease of the c-Myc expression level was due to the decreased β-catenin expression level in the cytoplasm. The E-cadherin/catenin complex, formed by β-catenin and E-cadherin, was significant in maintaining the structural integrity of the epithelial cells, inhibiting the migration of carcinoma cells and metabolism. It was also found that the SET expression level was significantly increased subsequent to knocking-down the FBXL20 expression level in the colon cell lines (9). Additionally, Amold *et al* (10) identified that protein phosphatase-2A (PP2A) dephosphorylates Axin, which leads to the destabilization and degradation of Axin. In our previous study, SET expression was observed to be upregulated, whereas PP2A expression was downregulated (9). Therefore, the reduced level of PP2A resulted in a low level of β-catenin due to an accumulation of the Axin-adenomatous polyposis coli (APC)-casein kinase 1-GSK3β complex.

To the best of our knowledge, there are no studies regarding human colorectal adenocarcinoma that identify the biological activity of colon cancer cells or the mechanism of FBXL20 upregulation in the colon cell lines. The aim of the present study was to determine whether colon cancer cells, which overexpressed FBXL20, showed signs of an abnormal Wnt signaling pathway by measurement of the β-catenin, E-cadherin, SET, p53, caspase 3, PP2A, c-Myc and Axin expression levels, in addition, cell proliferation and migration ability were observed.

## Materials and methods

### Cell culture

Colorectal adenocarcinoma cell lines (Lovo, SW480, SW620, Ls174T, HCT116 and HT29) were purchased from the American Type Culture Collection (Manassas, VA, USA). The colorectal cancer (CRC) cell lines were cultured in Dulbecco’s modified Eagle’s medium supplemented with 10% heat-inactivated fetal bovine serum.

### Plasmid construction and cell transfection

The oligonucleotides containing the whole coding DNA sequences of the FBXL20 gene were synthesized through a chemical method (Sangon Biotech, Shanghai, China). The annealed complementary oligonucleotides were subsequently inserted into the *Bam*H1/*Bbs*I site of the pReceiver-M03 expression plasmid. The engineered pReceiver-M03-FBL20 expression plasmid was verified by restriction digestion and sequenced by the Beijing Genomic Institute (Beijing, China). The recombinant pReceiver-M03-negative control (NC) vector was obtained as described above, however, the inserted sequence was not homologous with any human gene. The Lovo cells were transfected with 1 μg pReceiver-M03-FBL20 expression plasmid according to the manufacturer’s instructions (Lipofectamine 2000; Invitrogen Life Technologies, Carlsbad, CA, USA).

### Total RNA and protein extraction

Total RNA was extracted from the colorectal adenocarcinoma cells by the TRIzol reagent (Invitrogen Life Technologies) and purified with RNeasy columns (Tiangen Biotech Co., Ltd,, Beijing, China). An RNase-Free DNase kit (Takara Bio., Inc., Otsu, Shiga, Japan) was used to digest DNA. Cells were lysed with radioimmunoprecipitation assay buffer (Beyotime Co., Shanghai, China) containing protease inhibitors (Sigma-Aldrich, St. Louis, MO, USA) and protein quantification was conducted using a bicinchoninic acid protein assay kit (Beyotime Co.).

### Proliferation assays

The proliferation assay was performed 24 h after transfecting the pReceiver-M03-FBL20 expression plasmid into a 96-well plate by the addition of 10 μl cell counting kit 8 (CCK8; Sigma-Aldrich) into the medium, followed by incubation for 30 min under normal cell culture conditions. Cell viability was measured by absorbance at 450 nm using a microplate reader (Bio-Rad, Hercules, CA, USA).

### Cell invasion and wound healing assay

Cell invasion assays were performed with the 24-well cell invasion assay kit (Sigma-Aldrich) according to the manufacturer’s instructions. Cells (n=900) were resuspended in culture medium without FBS and placed in the upper Transwell chamber in triplicate. After a 24-h incubation at 37°C (5% CO_2_ atmosphere), the cells in the upper surface of the membrane were removed using a cotton swab and the cells that had migrated to the lower surface of the membrane were fixed and stained. The migrated cells on the lower surface of the membrane filter were scored from six random fields under a CKX31 microscope (magnification, ×400; Olympus, Tokyo, Japan). The results are shown as percentage migrating rate (%): % = (migrated cells/total cells) × 100. The wound healing assay was performed according to the manufacturer’s instructions. To generate a wound field, cells were cultured until they formed a monolayer around the insert. Subsequent to removing the insert, an open wound field was generated and cells were able to migrate from either side of the gap.

### Quantitative polymerase chain reaction (qPCR)

qPCR was performed in triplicate with a Mastercycler^®^ ep realplex system (Eppendorf, Hamburg, Germany) using the SYBR Premix Ex Taq mix (Takara) according to the manufacturer’s instructions. The primers were purchased from Sangon Biotech. GAPDH served as an endogenous control.

### Western blot analysis

The total cell protein (50 μg) was used for western blotting. The samples were resolved in 10% SDS-PAGE gels and transferred to polyvinylidene fluoride membranes. The membranes were immersed in western blocking buffer (Beyotime Co.) for 1 h and probed with the primary polyclonal antibody against FBL20, E-cadherin, SET, Axin, PP2A, p53, caspase 3 and β-actin (Santa Cruz Biotechnology, Inc., Santa Cruz, CA, USA) overnight at 4°C. The blots were washed in tris-buffered saline containing 0.1% Tween-20 and labeled with horseradish peroxidase-conjugated secondary anti-rabbit antibody (Santa Cruz Biotechnology, Inc.). Proteins were enhanced by BeyoECL Plus (Beyotime Co.) for visualization. The protein expression levels were expressed relative to β-actin levels.

### Statistical analysis

The results presented in the current study are the means ± standard error of the mean. Statistical analysis was performed by Student’s t-test, Fisher’s exact probability test and analysis of variance with SPSS 17.0 software (SPSS, Inc., Chicago, IL, USA). P<0.05 was considered to indicate a statistically significant difference.

## Results

### FBXL20 expression is significantly decreased in the Lovo cell line

**Figure 1:**
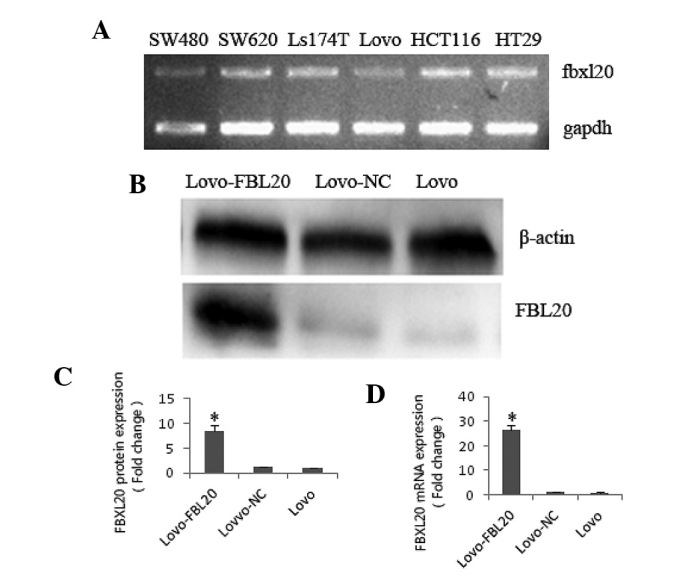
mRNA and protein expression of FBXL20 as detected by quantitative polymerase chain reaction and western blotting, respectively. (A) The FBXL20 expression level in Lovo cells is significantly lower compared with the SW480, SW620, Ls174T, HCT116 and HT29 cells. (B) The pReceiver-M03-FBL20 expression plasmid specifically upregulated the FBXL20 expression in Lovo-FBL20 cells. (C) The protein expression level of FBXL20 was increased by the pReceiver-M03-FBL20 expression plasmid. (D) The mRNA expression level of FBXL20 was also increased by the pReceiver-M03-FBL20 expression plasmid. mRNA and protein were extracted two days after transient transfection with the proper mixture of pReceiver-M03-FBL20 expression plasmid and Lipofectamine 2000. GAPDH and β-actin were used for normalization of mRNA and protein loading, respectively. The results were expressed as the mean ± standard deviation for three independent experiments. ^*^P<0.05 vs. Lovo cels, as determined by analysis of variance test. NC, negative control vector.

FBXL20 expression levels in the CRC cell lines (five types) were identified by qPCR. It was shown that the FBXL20 expression level was significantly lower in the Lovo cell line compared with the SW480, SW620, Ls174T, HT29 and HCT116 cell lines (Fig. 1A).

### FBXL20 expression level in the Lovo-FBL20 cell line

To investigate the function of FBXL20, the pReceiver-M03-FBL20 expression plasmid, which overexpressed the FBXL20 gene, was transfected into the Lovo cell line. At 48 h after transfecting the pReceiver-M03-FBL20 expression plasmid or pReceiver-M03-NC into the Lovo cells, the FBXL20 expression level was verified by qPCR (fold change, 27.392) and western blotting (fold change, 8.43), compared with that in the Lovo cells (Fig. 1B–D).

### Effect of FBXL20 overexpression on cell viability and migration in the Lovo-FBL20 cell line

**Figure 2:**
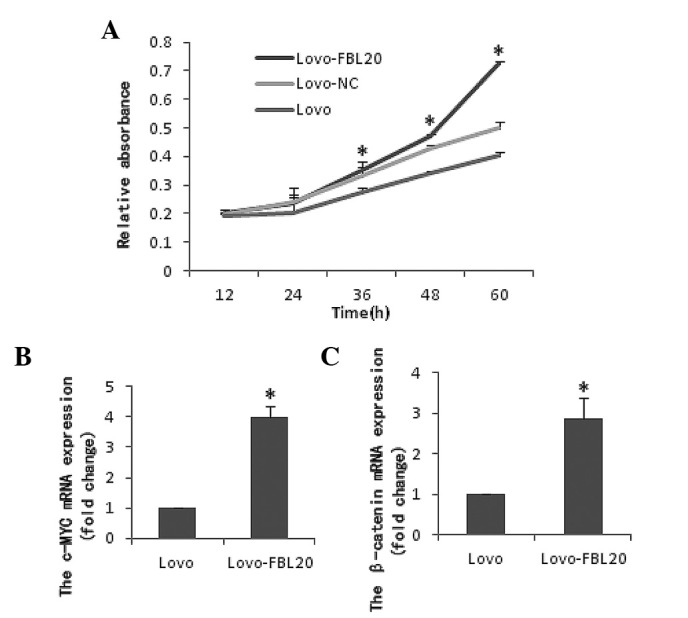
Cell proliferation effect of FBXL20 overexpressed in colorectal adenocarcinoma Lovo cells. (A) Cell viability assay. Human colorectal adenocarcinoma cells were incubated for the indicated time-periods. As a measurement of cell viability, the relative absorbances (means ± SD; n=6), obtained from the CCK8 assay, are shown. The mRNA expression level of (B) c-Myc and (C) β-catenin were upregulated by the pReceiver-M03-FBL20 expression plasmid vector in Lovo-FBL20 cells. RNA was extracted two days after transient transfection with the proper mixture of pReceiver-M03-FBL20 expression plasmid and Lipofectamine 2000. GAPDH was used for normalization of the RNA. The results were expressed as means ± SD for three independent experiments. ^*^P<0.05 compated with the Lovo cells, as determined by Student’s t-test. NC, negative control vector; CCK-8, cell counting kit 8; SD, standard deviation.

**Figure 3:**
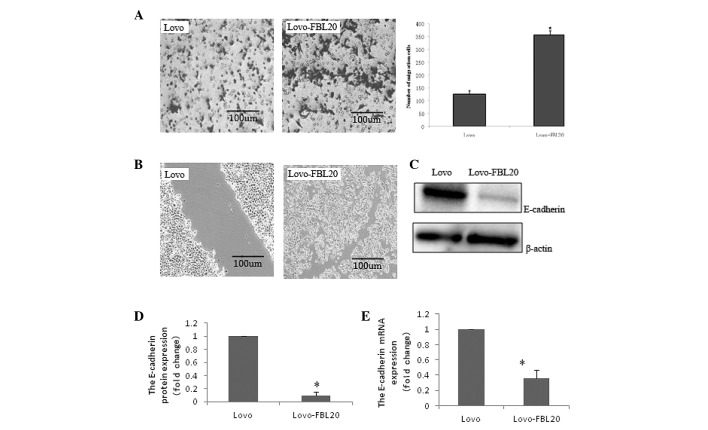
Cell invasion effect of FBXL20 overexpression on colorectal adenocarcinoma Lovo cells. (A) Transwell chamber assay (20 h). Human colorectal adenocarcinoma Lovo cells were incubated in the upper chamber subsequent to transfecting the pReceiver-M03-FBL20 expression plasmid into the cells. As a measurement of cell invasion, the cells in the lower membrane were counted (means ± SD; n=6) as shown. (B) Wound healing assay (24 h). (C) Protein expression using immunoblotting of whole proteins from the Lovo-FBL20 and Lovo cells. E-cadherin expression levels were significantly downregulated in Lovo-FBL20 cells compared with those of the Lovo cells. Representative blots of E-cadherin and β-actin expression levels. The (D) protein and (E) mRNA expression levels of E-cadherin were significantly downregulated by the pReceiver-M03-FBL20 expression plasmid in Lovo-FBL20 cells. mRNA was extracted two days after transient transfection with the proper mixture of pReceiver-M03-FBL20 expression plasmid and Lipofectamine 2000. GAPDH and β-actin were used for normalization of the mRNA and protein. The results were expressed as means ± SD for three independent experiments. ^*^P<0.05 indicates a significant increase compared with the control value. SD, standard deviation.

Cell viability (CCK8 assay) and cell invasion (Transwell chamber and wound healing assays) were performed using the Lovo cells that were transiently transfected with the pReceiver-M03-FBL20 expression plasmid. It was observed that the cell proliferation and migration activity was significantly increased in the Lovo-FBL20 cells compared with that of the Lovo cells. The percentages for the migrating rates in Lovo-FBL20 and Lovo cells were 42.18% (37.9±85.28%) and 14.05% (12.6±16.05%), respectively (data not shown). The difference between the Lovo-FBL20 and Lovo cells was identified to be statistically significant (P=0.007; Figs. 2 and 3).

### Effect of FBXL20 overexpression on the expression of E-cadherin, SET, pp2a, Axin, β-catenin, c-Myc, p53 and caspase 3 in the Lovo-FBL20 cells

To investigate the function of FBXL20, specific molecules were detected in the Lovo-FBL20 cells by qPCR and western blotting. qPCR and western blotting demonstrated that the mRNA expression and protein expression level of E-cadherin in the Lovo-FBL20 cells was significantly reduced compared with that in the Lovo cells, and the reduction rates were 63.50 and 90.20%, for mRNA and protein expression, respectively, which was considered to be statistically significant (P=0.001 and P<0.001; Fig. 3)

**Figure 4:**
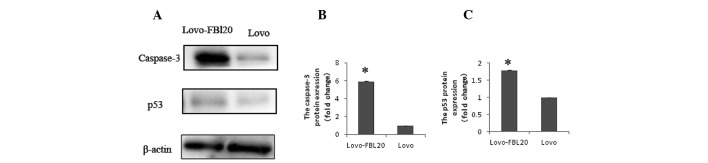
Protein expression using immunobloting of whole proteins from the Lovo-FBL20 and Lovo cells. Caspase 3 and p53 protein expression levels were significantly upregulated in Lovo-FBL20 cells compared with those of the Lovo cells. (A) Representative blots of caspase 3, p53 and β-actin expression levels. (B) Average signal intensity values plotted for caspase 3 protein expression and (C) p53 protein expression. 50 μg protein loaded per lane. All signals were normalized to β-actin. Values are expressed as means ± SD for three independent experiments. ^*^ P<0.05 indicates a significant increase compared with the control value. SD, standard deviation.

The mRNA levels of β-catenin and c-Myc in the Lovo-FBL20 cells were significantly upregulated compared with the Lovo cells. The β-catenin and c-Myc expression levels in the Lovo-FBL20 cells were 2.870 and 3.997-fold, respectively, compared with that in the Lovo cells (P<0.001 and P<0.001; Fig. 2). Additionally, it was found that the p53 and caspase 3 expression levels were significantly increased in the Lovo-FBL20 cell line. The p53 and caspase 3 expression levels in Lovo-FBL20 were 1.780 and 5.892-fold, respectively, compared with that in the Lovo cells, which was identified to be a statistically significant difference (P=0.007 and P<0.001; Fig. 4).

**Figure 5:**
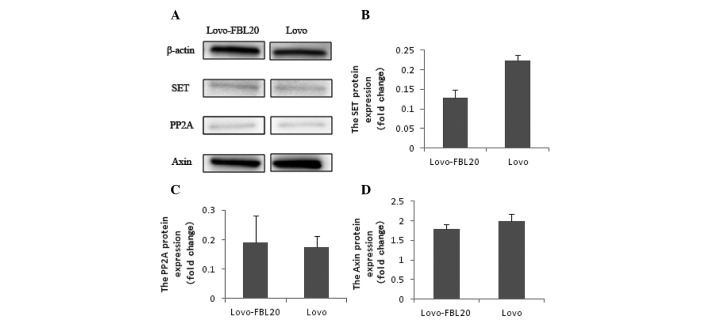
Protein expression using immunobloting of whole proteins from the Lovo-FBL20 and Lovo cells. No statistically significant differences in the expression levels of SET, PP2A and Axin proteins were identified between the Lovo-FBL20 and Lovo cells. (A) Representative blots of SET, PP2A and Axin expression levels. Average signal intensity values are plotted for (B) SET, (C) PP2A and (D) Axin protein expression. 50 μg protein loaded per lane. All signals were normalized to β-actin. Values are expressed as means ± SD for three independent experiments. ^*^ P<0.05 indicates a significant increase compared with the control value. SET, SET nuclear oncogene; PP2A, protein phosphatase-2A; SD, standard deviation.

However, the protein expression levels of SET, PP2A and Axin were not found to be significantly different between the Lovo-FBL20 and Lovo cells by western blot analysis (Fig. 5).

## Discussion

CRC remains a predominant cause of cancer mortality worldwide. Numerous studies have documented the significant roles of the F-Box family in the initiation of CRC. Grim *et al* (11) identified that the simultaneous deletion of Fbw7 and p53 resulted in highly penetrant, aggressive, metastatic adenocarcinoma, and allografts that originated from these tumors became highly malignant adenocarcinoma. In another study, FWD1, an F-box/WD40-repeat protein, was shown to form a specific multimolecular complex with β-catenin, Axin, GSK-3β and APC. The level of β-catenin in the cytoplasm was decreased by FWD1-facilitated ubiquitination and degradation of β-catenin (12). In addition, the E-cadherin and SET expression levels were markedly upregulated when the FBXL20 gene was knocked out in colorectal adenocarcinoma. However, no studies were found concerning the association between FBXL20, E-cadherin and SET.

In the present study, cell proliferation activity was significantly increased in the Lovo-FBL20 cells, compared with that of the corresponding control cells. The selective ubiquitination of proteins by ubiquitin E3 ligases exhibits a significant regulatory role in the control of cell differentiation, growth and transformation, and the dysregulation of ubiquitin E3 ligases is often associated with pathological outcomes, including tumorigenesis (13). Cell proliferation was notably reduced following inhibition of RNF5 (a ubiquitin E3 ligase) expression and resulted in reorganization of the actin cytoskeleton in reaction to the stress in MCF-7; however, this was not observed in p53 mutant breast cancer cells, indicating a p53-dependent function (14). Glycoprotein 78 (gp78) is a cell surface receptor that also functions as an E3 ubiquitin ligase in the endoplasmic reticulum-associated degradation pathway. A study by Tsai *et al* (15) reported that the expression of gp78 exhibits a causal role in the metastasis of an aggressive human sarcoma. Furthermore, gp78 is associated with, and targets, the transmembrane metastasis suppressor, KIA1, for degradation. Suppression of gp78 increases the abundance of KIA1 and reduces the metastatic potential of tumor cells (15). Based on the observation of the present study, it is speculated that the increased proliferation and migration abilities of the Lovo-FBL20 cells resulted from the upregulated expression level of FBXL20.

The mechanism by which the upregulated FBXL20 expression level leads to the increased proliferation of the colorectal adenocarcinoma cells remains unknown. The c-Myc and β-catenin expression levels in the present study were significantly upregulated in the Lovo-FBL20 cells compared with those in the corresponding control cells. Numerous studies have confirmed that the development of CRC was closely correlated with the activation of the Wnt signaling pathway (9,16,17). Furthermore, the activated Wnt signaling pathway was determined by the expression level of β-catenin in the cytoplasm as well as c-Myc. In addition, the c-Myc expression level was correlated with the proliferation capacity of neoplastic cells (18). Stimulation of the Wnt/β-catenin and Ras oncogene by P21-activated kinase 1 increases the progression of CRC and enhances survival by the stimulation of hypoxia-inducible factor 1α and β-catenin (19). In another study, Wu *et al* (20) showed that β-catenin increased T-cell factor 4 (TCF4) transcription activity and expression of its target genes, including cyclin D1 and c-Myc, in CRC cells. Correlation analysis showed that β-catenin expression in CRC tissues was positively associated with c-Myc expression. These findings raised the possibility that the increased proliferation capacity of the Lovo-FBL20 cells was due to the upregulation of c-Myc expression, as a consequence of the activation of the Wnt signaling pathway caused by accumulation of β-catenin in the cytoplasm.

A notable finding of the present study is that the E-cadherin expression level was significantly reduced and the migration ability of the Lovo-FFBL20 cells was significantly increased, which indicated that the migration ability was correlated with the E-cadherin expression level. These data also support the recent observation that downregulation of E-cadherin in pancreatic adenocarcinoma may promote invasion and metastasis of cancer cells (21). In addition, another study showed that induced expression of NFATc1CA downregulated E-cadherin expression and increased the invasive activity in tumor xenografts *in vivo* (22). Recent studies have shown that numerous E3 ubiquitin ligases, including Cb1, smurf1, BCA2 and SCF(β-TRCP), are critical in cell adhesion and migration (21). Cell adhesion is negatively regulated by Cb1 via ubiquitination of α integrin and Rap1, and inhibits actin polymerization by ubiquitination of mDab1 and WAVE2, while SCF(β-TRCP) ubiquitinates Snail (a transcriptional repressor of E-cadherin) to inhibit cell migration. All of the aforementioned studies are consistent with the results of the current study which identified that the FBXL20 mRNA and protein expression was markedly higher in the Lovo-FBL20 cells than the control Lovo cells, and the migration rates of the Lovo-FBL20 cells were significantly higher than those of the Lovo cells. Therefore, we hypothesize that the higher migration rates are associated with the higher FBXL20 RNA and protein expression levels. It is well known that E-cadherin expression negatively correlates with migration activity, and FBXL20 is a type of E3 ubiquitin ligase, which functions via ubiquitin in specific cells. Therefore, we suspect that FBXL20 may be involved in the ubiquitination of E-cadherin (23).

P53 is a well-known tumor suppressor gene whose loss of function emphasizes the most common genetic alteration in human malignancy. It is involved in DNA damage repair, cell cycle regulation, apoptosis, ageing and cellular senescence (18). In the present study, the p53 and caspase 3 expression levels were significantly increased in the Lovo-FBL20 cells. Li *et al* (24) reported that the Wnt signaling pathway regulator, Axin, regulates p53-dependent apoptosis in response to DNA damage. However, in another study, it was shown that Axin downregulates TCF4 transcription via β-catenin, independently of p53, and that Axin may inhibit the proliferation and invasion of lung cancer cells via β-catenin (25). In addition, in the present study, there was no statistical difference identified between the Lovo-FBL20 and Lovo cells with regard to the Axin level. Thus, Axin may upregulate caspase 3 transcription independent of p53.

In the present study, SET expression was not found to be correlated with an increased FBXL20 expression in the Lovo-FBL20 cells. Although, it was shown that the SET expression level was markedly increased following silencing of the FBXL20 gene in the colorectal adenocarcinoma cell lines, SW480 and SW620. These findings support the results that the Axin level did not increase with the upregulation of p53 in the Lovo-FBL20 cells. PP2A is a multi*-*subunit serine/threonine phosphatase that is integral for intracellular signaling, cell cycle progression and gene regulation. Ikeda *et al* (26) reported that the heterodimeric form of PP2A directly binds to Axin, and subsequently Axin is dephosphorylated and induced into the ubiquitin degradation process. In another study, SET has been shown to be a natural inhibitor of the PP2A protein (27). Notably, the marked difference in PP2A expression level between the Lovo-FBL20 and Lovo cells was not found in the present study. These novel findings highlight that FBXL20 does not participate directly in the ubiquitin process of SET.

In conclusion, the present data on the roles of FBXL20 in colorectal adenocarcinoma cells are comparable with other F-Box family members, which mediate the specific substrate protein into the ubiquitin proteasome pathway. The present study indicated that FBXL20 may mediate the ubiquitin degradation of E-cadherin resulting in an increased invasive ability of malignant cells. However, further studies are required to identify all of the factors that are involved in the FBXL20-mediated ubiquitin proteasome process in colorectal adenocarcinoma.
